# An epidemiological study on skin tumors of the elderly in a community in Shanghai, China

**DOI:** 10.1038/s41598-023-29012-1

**Published:** 2023-03-17

**Authors:** Jianhua Huang, Linglin Zhang, Lei Shi, Minfeng Wu, Ting Lv, Yunfeng Zhang, Yongxian Lai, Qingfeng Tu, Xiuli Wang, Hongwei Wang

**Affiliations:** 1 Department of Dermatology, Huadong Hospital, Fudan University, Shanghai, China; 2 Institute of Photomedicine, Shanghai Skin Disease Hospital, Tongji University School of Medicine, Shanghai, China

**Keywords:** Cancer, Health care, Medical research, Risk factors

## Abstract

The morbidity of skin tumors (ST) in China is a great concern as the population ages. No epidemiological survey on ST in elderly communities in China has been reported. A questionnaire survey was conducted among the residents over 60 years old in a community in Shanghai, China from May 1, 2011 to November 30, 2011. The prevalence of cutaneous tumors and associated factors were analyzed. Among 2038 valid cases, a total of 78 (3.8%, 95% CI 3.0–4.7) skin cancers (SC) were confirmed. According to the final multivariate regression analysis, age, gender and previous occupation were the significantly influential factors for SC. Actinic keratosis (AK) accounted for the largest proportion (63, 3.1%) in SC. The head and neck was the physiological site with the highest incidence of SC (64, 82.1%), and AK was the most common (55, 87.3%) in head and neck SC. The common concomitant diseases of SC were hypertension (26, 33.3%) and diabetes mellitus (9, 11.5%). Seborrheic keratosis (SK) was the most common benign skin tumor with a prevalence of 100%. Men and women developed SK in significantly different parts of the body (*P* < 0.0001). The incidence of ST in the elderly population in Shanghai community increased with age. ST preferred to occur in the head and neck, which might be attributed to excessive ultraviolet (UV) exposure in these areas. Therefore, early diagnosis and sun-protection education are essential interventions for ST in the elderly.

## Introduction

SC is diagnosed more commonly than other malignancies and is one of the most distressing fatal skin diseases, especially in the elderly^1–4^. It poses an enormous global public health burden on socioeconomic and medical costs^5^. During 2007–2011, approximately 5 million adults received SC treatment in the U.S. each year, resulting in an average annual cost of $8.1 billion^6^. An estimation of the total direct, indirect and intangible costs per basal cell carcinoma (BCC) in Canada in 2011 was $4312.9^7^. In addition to the medical costs, melanoma was responsible for the majority of SC deaths, accounting for approximately 9000 annual deaths in the U.S.^8^. These findings underlined that the health and economic burden of SC was substantial and its incidence continued to be high over the past decades^9^. Therefore, an epidemiological investigation on the prevalence of ST and related high-risk factors is considered to be significant for ST control and prevention measures.

Besides the above worrying figures from Caucasians, the condition of ST in China is also a great concern with changes in lifestyle, ozone layer destruction and aging^10^. However, it may exhibit diverse features and prognosis from western countries. Previous epidemiological studies on tumors in China was limited to some highly lethal malignancies, such as lung cancer or gastrointestinal cancer^11–13^. To date, there is a lack of comprehensive survey on the prevalence and risk factors of SC in elderly communities in China. The aim of the this study is to elucidate the epidemiology of SC in urban elderly communities by exploring the regularity of SC in an elderly community in Shanghai, China.

## Methods

### Study settings

An observational, cross-sectional prevalence study was conducted with an epidemiological cluster sampling questionnaire among elderly residents in a community in Shanghai, China from May 1, 2011 to November 30, 2011. This study was approved by the ethics committee of Shanghai skin disease hospital affiliated to Tongji University. All methods were performed in accordance with the relevant guidelines and regulations of Declaration of Helsinki.

### Sampling and sample size

We selected a mature community with typical aging characteristics in Shanghai as the research object, which was fully able to reflect the status of ST among the elderly in the city and was highly representative. The sampling framework was based on the elderly population for Shanghai in 2011. The projected sample size was expected to be 385 based on a margin of error of 5% and 95% confidence interval with an estimated 50% response rate^14^. Actually, almost the entire community of seniors was studied, so the sample size exceeded the calculated size. Such an expansion of sample size did not diminish the accuracy of our final statistical results.

### Inclusion and exclusion criteria

All permanent residents over the age of 60 who had resided in the area for more than 6 months were eligible, with the exception of elderly residents in nursing homes and hospitals. Those that refused to supply true information, were unwilling to fill in informed consent, and had poor compliance should be excluded. Written informed consent was obtained from the participants.

### Data extraction and quality assessment

Our survey involved basic information, categories of SC, risk factors and concomitant diseases, etc. To minimize bias, each eligible participant was randomly selected and underwent dermatological examinations by three board-certified dermatologists according to standard procedures. The dermatologists underwent special training and technical assessment, and all of them were qualified as experienced specialists in the field of skin cancer before this project. Data were extracted through questionnaires and dermatological examinations. Unified forms, methods, form filling instructions and diagnostic criteria were adopted. The elderly were summoned to the residential committee for examination. Door-to-door inspection was provided for those with mobility difficulties. It usually took 10–20 min to complete the medical history inquiry and dermoscopy of each subject’s skin lesions. All the completed forms were checked and verified by the quality control personnel. The data were recorded and checked twice by different personnel. Most lesions could be clinically diagnosed, while for highly suspicious lesions, pathological diagnosis was performed prior to statistical analysis. Diagnosis was based on the International Classification of Diseases (ICD-10). We collectively referred to malignant melanoma (MM), squamous cell carcinoma (SCC), BCC, AK, keratoacanthoma (KA) and cutaneous horn (CH) with malignant cells as SC, and the rest as benign ST including SK and skin tags. All the screened lesions were diagnosed for the first time.

### Statistics

All categorical variables were summarized as percentages, and continuous variables were represented as mean ± standard deviation (SD). Inter-group comparisons were performed using the chi-square or Fisher’s exact test. Univariate regression analysis preliminarily screened the influence of various independent factors on SC prevalence, and multivariate regression analysis was used to further eliminate the interference of confounding factors, making the statistical results more reliable. SPSS (Version 24, IBM Corporation, New York, NY) was employed for all statistical work. Odds ratios (OR) and 95% CIs were calculated, with a CI excluding 1.00 considered statistically significant. Graphics were produced with GraphPad Prism 8.0 (GraphPad Software Inc., San Diego, CA, USA). *P* < 0.05 was considered statistically significant.

## Results

### General characteristics

A total of 2082 questionnaires were distributed to the elderly aged over 60 years old and 2049 questionnaires were returned with a response rate of 98.41%. Finally, 2038 questionnaires were valid after eliminating 11 incomplete data of refusing physical examination. The average age was 72.05 ± 8.17 years old.

### Population composition ratio

The age group of ≥ 85 has the smallest population (106, 5.2%), while the 60–64 age group accounted for the largest population (524, 25.7%). The population of the other age groups fell between the above two groups. The number of female participants (1271, 62.4%) was nearly twice that of male participants (767, 37.6%). The overall education level was relatively high, with 203 (10.0%) having a college degree or above and 1030 (50.5%) having a secondary school degree. Type IV phototype represented the largest proportion (1395, 68.5%) of the elderly population, followed by type III (527, 25.9%) and type II (38, 1.9%). The populations of I, V and VI phototypes were quite scarce. The majority manifested type 3 (1623, 79.6%) photoaging, followed by type 4 (283, 13.9%), type 2 (124, 6.1%) and type 1 (8, 0.4%). 47.1% (959) residents worked indoors and only a minority (243, 11.9%) worked outdoors. Mixed jobs (836, 41.0%) meant working both indoors and outdoors. More than half of residents (1143, 56.1%) never adopted sun protection, while approximately a quarter (496, 24.3%) adopted frequently and even fewer adopted occasionally (399, 19.6%). The population proportion distribution of other factors can be referred to Table 1.

**Table 1:** Demographic characteristics of the elderly in the community, the prevalence of cutaneous malignancies.

Factors	Total, n (%)	Prevalence of Skin cancer, n (%)	Model 1	Model 2
N	2038	78 (3.8, 95% CI 3.0–4.7)		
Age (years) (Model 1***, Model 2**)				
60–64	524 (25.7)	8 (1.5, 95% CI 0.5–2.6)	Reference	Reference
65–69	291 (14.3)	7 (2.4, 95% CI 0.6–4.2)	1.590 (0.571–4.429)	1.325 (0.462–3.806)
70–74	309 (15.2)	7 (2.3, 95% CI 0.6–3.9)	1.495 (0.537–4.164)	1.251 (0.424–3.694)
75–79	511 (25.1)	30 (5.9, 95% CI 3.8–7.9)	4.023 (1.826–8.862)***	3.945 (1.575–9.880)**
80–84	297 (14.6)	15 (5.1, 95% CI 2.5–7.6)	3.431 (1.437–8.191)**	3.125 (1.109—8.807)*
≥ 85	106 (5.2)	11 (10.4, 95% CI 4.5–16.3)	7.468 (2.927–19.055)***	7.749 (2.478–24.232 )***
Sex (Model 1*, Model 2**)				
Male	767 (37.6)	20 (2.6, 95% CI 1.5–3.7)	Reference	Reference
Female	1271 (62.4)	58 (4.6, 95% CI 3.4–5.7)	1.786 (1.066–2.993)*	2.936 (1.490–5.787)**
Educational qualification (Model 1**)				
College degree or above	203 (10.0)	6 (3.0, 95% CI 0.6–5.3)	Reference	Reference
Secondary school	1030 (50.5)	25 (2.4, 95% CI 1.5–3.4)	0.871 (0.331–2.017)	0.771 (0.297–2.000)
Primary school	378 (18.6)	21 (5.6, 95% CI 3.2–7.9)	1.931 (0.767–4.865)	1.101 (0.394–3.076)
No school qualification	427 (21.0)	26 (6.1, 95% CI 3.8–8.4)	2.129 (0.862–5.257)	0.964 (0.329–2.822)
Marriage				
Married	1629 (79.9)	57 (3.5, 95% CI 2.6–4.4)	Reference	Reference
Unmarried	22 (1.1)	1 (4.5, 95% CI − 4.9–14.0)	1.313 (0.174–9.934)	0.916 (0.111–7.586)
Divorced	14 (0.7)	1 (7.1, 95% CI − 8.3–22.6)	2.121 (0.273–16.497)	1.878 (0.217–16.273)
Loss of spouse	373 (18.3)	19 (5.1, 95% CI 2.9–7.3)	1.480 (0.870–2.519)	0.625 (0.338–1.154)
Fitzpatrick skin type				
Light complexion	48 (2.4)	1 (2.1, 95% CI − 2.1–6.3)	Reference	Reference
I	10 (0.5)	0 (0.0, 95% CI 0.0–0.0)		
II	38 (1.9)	1 (2.6, 95% CI − 2.7–8.0)		
Medium Beige	1922 (94.3)	76 (4.0, 95% CI 3.1–4.8)	1.935 (0.263—14.211)	2.359 (0.303–18.386)
III	527 (25.9)	20 (3.8, 95% CI 2.2–5.4)		
IV	1395 (68.5)	56 (4.0, 95% CI 3.0–5.0)		
Dark complexion	68 (3.3)	1 (1.5, 95% CI − 1.5–4.4)	0.701 (0.043—11.499)	0.713 (0.041–12.445)
V	57 (2.8)	1 (1.8, 95% CI − 1.8–5.3)		
VI	11 (0.5)	0 (0.0, 95% CI 0.0–0.0)		
Degree of photoaging (Model 1**)				
Type 1	8 (0.4)	1 (12.5, 95% CI − 17.1–42.1)	Reference	Reference
Type 2	124 (6.1)	4 (3.2, 95% CI 0.1–6.4)	0.233 (0.023–2.374)	0.151 (0.012–1.910)
Type 3	1623 (79.6)	51 (3.1, 95% CI 2.3–4.0)	0.227 (0.027–1.880)	0.067 (0.006–0.726)*
Type 4	283 (13.9)	22 (7.8, 95% CI 4.6–10.9)	0.590 (0.069–5.015)	0.104 (0.009–1.183)
Hair				
Smooth and shiny	496 (24.3)	14 (2.8, 95% CI 1.4–4.3)	Reference	Reference
Thin	533 (26.2)	27 (5.1, 95% CI 3.2–6.9)	1.837 (0.952–3.545)	1.866 (0.888–3.920)
Yellow and forked	1009 (49.5)	37 (3.7, 95% CI 2.5–4.8)	1.311 (0.702–2.447)	1.152 (0.592–2.243)
Eye wrinkles				
Superficial wrinkles	172 (8.4)	2 (1.2, 95% CI − 0.5–2.8)	Reference	Reference
Medium depth wrinkles	1004 (49.3)	37 (3.7, 95% CI 2.5–4.9)	3.252 (0.777–13.620)	4.001 (0.827–19.346)
Deep wrinkles with clear edges	862 (42.3)	39 (4.5, 95% CI 3.1–5.9)	4.028 (0.963–16.841)	3.003 (0.602–14.979)
Hereditary skin history				
No	1985 (97.4)	76 (3.8, 95% CI 3.0–4.7)	Reference	Reference
Yes	53 (2.6)	2 (3.8, 95% CI − 1.5–9.1)	0.985 (0.235–4.122)	1.392 (0.318–6.092)
Epidermolysis bullosa	35 (66.0)	1 (2.9, 95% CI − 2.9–8.7)		
Vitiligo	6 (11.3)	0 (0.0, 95% CI 0.0–0.0)		
Psoriasis	5 (9.4)	0 (0.0, 95% CI 0.0–0.0)		
Xeroderma pigmentosum	1 (1.9)	0 (0.0, 95% CI 0.0–0.0)		
Lupus erythematosus	1 (1.9)	0 (0.0, 95% CI 0.0–0.0)		
Ichthyosis	1 (1.9)	0 (0.0, 95% CI 0.0–0.0)		
Seasonal dermatitis	1 (1.9)	1 (100.00)		
Others	4 (7.6)	0 (0.0, 95% CI 0.0–0.0)		
History of cataract (Model 1*)				
No	1255 (61.6)	39 (3.1, 95% CI 2.1–4.1)	Reference	Reference
Yes	783 (38.3)	39 (5.0, 95% CI 3.5–6.5)	1.634 (1.039–2.571)*	1.190 (0.725–1.951)
History of macular degeneration				
No	1986 (97.5)	75 (3.8, 95% CI 2.9–4.6)	Reference	Reference
Yes	52 (2.6)	3 (5.8, 95% CI − 0.8–12.3)	1.560 (0.475–5.119)	1.456 (0.422–5.023)
Previous profession (Model 2*)				
Outdoor work	243 (11.9)	15 (6.2, 95% CI 3.1–9.2)	Reference	Reference
Mixed work	836 (41.0)	31 (3.7, 95% CI 2.4–5.0)	0.585 (0.311–1.103)	0.544 (0.268–1.105)
Indoor work	959 (47.1)	32 (3.3, 95% CI 2.2–4.5)	0.525 (0.279–0.985)*	0.371 (0.188–0.731)**
Use of physical sun protection				
Never	1143 (56.1)	44 (3.8, 95% CI 2.7–5.0)	Reference	Reference
Frequently	496 (24.3)	17 (3.4, 95% CI 1.8–5.0)	0.886 (0.501–1.567)	0.998 (0.539- 1.848)
Occasionally	399 (19.6)	17 (4.3, 95% CI 2.3–6.3)	0.112 (0.628–1.969)	1.056 (0.574–1.943)
Smoke				
Never	1743 (85.5)	67 (3.8, 95% CI 2.9–4.7)	Reference	Reference
Frequently	218 (10.7)	7 (3.2, 95% CI 0.9–5.6)	0.830 (0.376–1.831)	1.763 (0.735–4.229)
Occasionally	77 (3.8)	4 (5.2, 95% CI 0.1–10.3)	1.371 (0.487–3.861)	2.179 (0.710–6.689)
Photosensitive food consumption history				
No	1629 (79.9)	64 (3.9, 95% CI 3.0–4.9)	Reference	Reference
Yes	409 (20.1)	14 (3.4, 95% CI 1.7–5.2)	0.867 (0.481–1.562)	0.932 (0.497–1.747)
Spinach	203 (49.6)	7 (3.4, 95% CI 0.9–6.0)		
Carrot	312 (76.3)	11 (3.5, 95% CI 1.5–5.6)		
Celery	339 (82.9)	10 (2.9, 95% CI 1.1–4.8)		
Marinated mud snail	16 (3.9)	0 (0.0, 95% CI 0.0–0.0)		
Mango	30 (7.3)	1 (3.3, 95% CI − 3.5–10.2)		
Chemical exposure history				
No	1792 (87.9)	70 (3.9, 95% CI 3.0–4.8)	Reference	Reference
Yes	246 (12.1)	8 (3.3, 95% CI 1.0–5.5)	0.827 (0.393–1.740)	1.159 (0.532–2.524)
Sunburn history				
Yes	60 (2.9)	2 (3.3, 95% CI − 1.3–8.0)	Reference	Reference
No	1978 (97.1)	76 (3.8, 95% CI 3.0–4.7)	1.159 (0.278–4.833)	1.299 (0.293–5.748)
Radiation or chemotherapy				
Yes	23 (1.1)	1 (4.3, 95% CI − 4.7–13.4)	Reference	Reference
No	2015 (98.9)	77 (3.8, 95% CI 3.0–4.7)	0.874 (0.116–6.569)	0.993 (0.115–8.573)

Odds ratios for associated potential risk factors were determined by univariable and multivariable logistic regression analyses. Model 1 represented univariate regression analysis, and Model 2 represented multivariate regression analysis. 95% CI = 95% confidence interval. *P < 0.05; **P < 0.01; ***P < 0.001.

### SC prevalence

A total of 78 cases of SC were confirmed, and the overall prevalence was 3.8% (95% CI 3.0–4.7%). The composition ratio displayed the prevalence of cutaneous malignancies dominated the higher age range. The highest SC prevalence rate (11, 10.4%, 95% CI 4.5–16.3%) emerged in the ≥ 85 age group, while the lowest was in the 60–64 age group (8, 1.5%, 95% CI 0.5–2.6%). The standardized prevalence rates of SC in 60–70, 70–80 and ≥ 80 age group was 954.8 (CI 944.5–965.0) per 100,000, 1355.1 (CI 1343.0–1367.3) per 100,000 and 1167.3 (CI 1156.0–1178.6) per 100,000, respectively, using the Shanghai elderly population as standard. Men (20, 2.6%, 95% CI 1.5–3.7%) appeared to be less likely to develop SC than women (58, 4.6%, 95% CI 3.4–5.7%). The standardized prevalence rates of SC for females and males were 2394.3 (CI 2378.3–1178.6) per 100,000, and 1239.6 (CI 1227.9–1251.2) per 100,000, respectively, using the Shanghai elderly population as standard. The percentages implied that those with primary (21, 5.6%, 95% CI 3.2–7.9%) and illiterate (26, 6.1%, 95% CI 3.8–8.4) education seemed to be more susceptible to skin neoplasms than those with secondary (25, 2.4%, 95% CI 1.5–3.4%) and college degrees (6, 3.0%, 95% CI 0.6–5.3%). In our investigation, the overall trend appeared that type IV (56, 4.0%, 95% CI 3.0–5.0%) was more susceptible to cutaneous tumors than type III (20, 3.8%, 95% CI 2.2–5.4%). The SC incidence was the lowest in those who worked indoors (32, 3.3%, 95% CI 2.2–4.5%), compared to those with mixed occupations (31, 3.7%, 95% CI 2.4–5.0%) and outdoor jobs (15, 6.2%, 95% CI 3.1–9.2%). Other percentages of SC prevalence were detailed in Table 1.

### Associated factors of skin malignancies

The preliminary screening of the risk factors of skin malignancies was conducted by univariate regression analysis in our survey. As can be seen in Table 1 (Model 1), univariate analysis showed that age (60–64 group: Reference; 65–69 group: OR 1.590, 95% CI 0.571–4.429; 70–74 group: OR 1.495, 95% CI 0.537–4.164; 75–79 group: OR 4.023, 95% CI 1.826–8.862; 80–84 group: OR 3.431, 95% CI 1.437–8.191; ≥ 85 group: OR 7.468, 95% CI 2.927–19.055), sex (Male: Reference; Female: OR 1.786, 95% CI 1.066–2.993), education (College degree or above: reference; Secondary school: OR 0.871, 95% CI 0.331–2.017; Primary school: OR 1.931, 95% CI 0.767–4.865; No school qualification: OR 2.129, 95% CI 0.862–5.257), photoaging (Type 1: Reference; Type 2: OR 0.233, 95% CI 0.023–2.374; Type 3: OR 0.227, 95% CI 0.027–1.880; Type 4: OR 0.590, 95% CI 0.069–5.015), cataract history (No: Reference; Yes: OR 1.634, 95% CI 1.039–2.571), and previous occupation (Outdoor work: Reference; Mixed work: OR 0.585, 95% CI 0.311–1.103; Indoor work: OR 0.525, 95% CI 0.279–0.985) exerted significant influence on the incidence of SC. As judged by the univariate analysis, none of the other factors we investigated exhibited any aggravating or protective effect on SC. After preliminary univariate analysis, the identification of confounders required further incorporation of the above factors into the multivariate regression model (Model 2). Age remained to be the most influential factor for SC (60–64 group: Reference; 65–69 group: OR 1.325, 95% CI 0.462–3.806; 70–74 group: OR 1.251, 95% CI 0.424–3.694; 75–79 group: OR 3.945, 95% CI 1.575–9.880; 80–84 group: OR 3.125, 95% CI 1.109–8.807; ≥ 85 group: OR 7.749, 95% CI 2.478–24.232). SC was found to be more prevalent in women than in men (Male: Reference; Female: OR 2.936, 95% CI 1.490–5.787). Although univariate analysis revealed the incidence of SC was inversely proportional to education, multivariate logistic data indicated that educational qualification was only a confounding factor in our study (College degree or above: reference; Secondary school: OR 0.771, 95% CI 0.297–2.000; Primary school: OR 1.101, 95% CI 0.394–3.076; No school qualification: OR 0.964, 95% CI 0.329–2.822). Similarly, the contribution of photoaging and cataract history to SC was also rendered negligible by multivariate analysis (Table 1). Multivariate logistic result turned out that indoor work was a protective factor and outdoor work was a risk for SC (Outdoor work: Reference; Mixed work: OR 0.544, 95% CI 0.268–1.105; Indoor work: OR 0.371, 95% CI 0.188–0.731). Apart from these factors, none of the other factors such as smoke, sunburn history and photosensitive food history showed any significant impact on SC (Table 1).

### Elaboration of the percentages of various SC

AK, SCC, BCC, Bowen's disease (BD), KA and CH were the six major categories of skin malignancies. Table 2 revealed that AK was the most common SC with a total of 63 (3.1%) cases, while the other epithelial neoplasms were relatively seldom. There were 3 (0.1%) cases of SCC, 9 (0.4%) cases of BCC, and 1 (0.0%) case of BD, KA and CH, respectively. No malignant tumors such as melanoma, mycosis fungoides, Paget’s diseases were detected. The prevalence rates of AK in 75–79 (26, 5.1%), 80–84 (13, 4.4%) and ≥ 85 (8, 7.5%) age groups were significantly higher than that in 60–64 (5, 0.9%) age group (*P* < 0.05).

**Table 2:** Prevalence of various cutaneous malignancies in different age groups.

Age group	Surveyed population	AK	SCC	BCC	BD	KA	CH
60–64	524	5 (0.9)	0	0	0	0	0
65–69	291	5 (1.7)	0	1 (0.3)	0	0	0
70–74	309	6 (1.9)	1 (0.3)	2 (0.6)	0	0	0
75–79	511	26 (5.1)	1 (0.2)	4 (0.8)	0	0	0
80–84	297	13 (4.4)	1 (0.3)	2 (0.7)	1 (0.3)	1 (0.3)	0
≥ 85	106	8 (7.5)	0	0	0	0	1 (0.9)
Sum	2038	63 (3.1)	3 (0.1)	9 (0.4)	1 (0.0)	1 (0.0)	1 (0.0)

*AK* Actinic keratosis, *SCC* Squamous cell carcinoma, *BCC* Basal cell carcinoma, *BD* Bowen's disease, *KA* Keratoacanthoma, *CH* Cutaneous horn.

The incidence of cutaneous neoplasms varied widely across body sites. Statistics revealed that ST occurred most frequently in the head and neck, up to 82.1% (64), followed by hands and feet 6.4% (5), limbs 6.4% (5), trunk 5.1% (4), and perineum and mucosa almost no ST. Compared with other parts, AK was prone to distribute in the head and neck (87.3%), much higher than that in the limb (3.2%) and trunk (1.6%), and its distribution percentage was comparable to that of BCC (66.7% in the head, 11.1% in the trunk and 22.2% in the body). Perhaps due to the limited sample size, only 1 case of BD was detected in the trunk in our survey. Intriguingly, SCC distribution rate was more uniform (33.3% for the limb, trunk and head) (Table 3).

**Table 3:** Analysis of the location of various skin cancers.

Skin cancer	Perineum and mucosa	Hand and foot	Limb	Trunk	Head and neck
AK	0	5 (7.9)	2 (3.2)	1 (1.6)	55 (87.3)
SCC	0	0	1 (33.3)	1 (33.3)	1 (33.3)
BCC	0	0	2 (22.2)	1 (11.1)	6 (66.7)
BD	0	0	0	1 (100.00)	0
KA	0	0	0	0	1 (100.0)
CH	0	0	0	0	1 (100.0)
Sum	0	5 (6.4)	5 (6.4)	4 (5.1)	64 (82.1)

*AK* Actinic keratosis, *SCC* Squamous cell carcinoma, *BCC* Basal cell carcinoma, *BD* Bowen's disease, *KA* Keratoacanthoma, *CH* Cutaneous horn.

The large proportions of AK were found in type III (16, 3.0%) and IV (46, 3.3%), and BCC was second to AK, accounting for 0.4% and 0.5% in type III and IV respectively. Confusingly, SC seemed not to occur in type I and VI. However, these two skin phototypes should not be included in the statistics due to too small population with these complexions in China (Table 4).

**Table 4:** The distribution of various skin malignancies in skin phototypes of the elderly.

Skin cancer	I	II	III	IV	V	VI
AK	0	1 (2.6)	16 (3.0)	46 (3.3)	0	0
SCC	0	0	1 (0.2)	2 (0.1)	0	0
BCC	0	0	2 (0.4)	7 (0.5)	0	0
BD	0	0	0	1 (0.0)	0	0
KA	0	0	1 (0.2)	0	0	0
CH	0	0	0	0	1 (1.8)	0
Sum	0	1 (2.6)	20 (3.8)	56 (4.0)	1 (1.8)	0

*AK* Actinic keratosis, *SCC* Squamous cell carcinoma, *BCC* Basal cell carcinoma, *BD* Bowen's disease, *KA* Keratoacanthoma, *CH* Cutaneous horn.

### Comorbidities

The common systemic comorbidities of SC and their percentages were elaborated in Supplementary Table S1. Among the 78 SC cases, 47 (60.3%) cases were accompanied by comorbid diseases: hypertension (26, 33.3%), diabetes mellitus (9, 11.5%), rheumatoid arthritis (4, 5.1%), psoriasis (4, 5.1%), chronic obstructive pulmonary disease (3, 3.8%) and urticaria (1, 1.3%) (Supplementary Table S1).

### Benign ST

SK (2038, 100.0%) was identified as the most common benign skin tumor. Body sites under chronic UV exposure, such as hand back (85.3%), temporal (80.4%), cheek (71.0%), were found to be prone to SK. Significant gender difference in the distribution of SK body parts was observed by chi-square test (*P* < 0.0001) (Supplementary Table S2 and Supplementary Fig. S1). Supplementary Fig. S2 depicted an ascending tendency that the average SK number was roughly proportional to the rise of age. Except SK, there were 67 (3.3%, 95% CI 2.5–4.1%) cases of other benign ST, including 21 (2.7%, 95% CI 1.6–3.9%) males and 46 (3.6%, 95% CI 2.6–4.6%) females, with no difference in gender distribution (*P* = 0.3407). No statistical difference was found in age group stratification of benign tumors except SK (*P* = 0.6673) (Supplementary Table S3).

## Discussion

SC is the most frequently diagnosed cancer in white populations and numerous studies have demonstrated that incidence of SC is ascending worldwide^15^. The highest prevalence of SC globally has been reported to be in New Zealand and Australia^16^. Nearly 50-fold and 100-fold differences in the frequency of BCC and SCC respectively occurred between Caucasian populations in northern Europe and Australia^17,18^. So far, the SC epidemiology in the elderly has not been well reported in Shanghai, China. After our representative survey, we concluded that the prevalence of elderly SC was 3.8%, which was significantly lower than that recorded in western countries. This discrepancy might lie not only in ethnic differences, but also in the fact that we mainly investigate the elderly population. As skin malignancies would result in a poor prognosis once they progressed, the necessity of SC cognition rendered this study the focus. This pioneer study was the first to describe the epidemiology of ST in an elderly community in Shanghai.

Since the morbidity of ST was considered to be in association with senescence, it was summarized from the perspective of age stratification in first. Our results found that cutaneous malignancies manifested an growth trend with age, with a substantial increment in the groups over 75 years old. The propellant role that age played in the morbidity of SC had also been supported by other studies^19–21^. Previous studies in western countries documented that more than 80% of skin neoplasms occurred in the elderly over 60 years old^22,23^. Approximately 53% of SC-related deaths occurred in persons over 65 years old^24^. The susceptibility of the elderly to epithelial neoplasms might be due to cumulative exposure to UV, decreased melanocyte density and immune senescence^20,25^.

Gender is also a key factor in the development of SC. Men's characteristics of physical work, outdoor lifestyle and neglect of sun protection all support the higher prevalence of epidermal tumors in men^26,27^. On the contrary, here we discovered females were more likely to develop cutaneous malignancies than males. An important explanation might be that in the last century, Shanghai’s labor force was mainly engaged in industrial production. A large number of young people might work in factories with occupational hazards, such as ultraviolet radiation. Also, this phenomenon might be attributed to women’s longer longevity compared with men and larger constituent of women in elderly community, as evidenced by the negative correlation between aging and the proportion of the male population (not shown in the table). The deviation in the distribution of SC between the sexes in our findings seemed to be inconsistent with the previous conclusions, which was recognized as a reflection of the particularity of SC in the elderly rather than a contradiction. What's more, further analysis of the interaction of various factors demonstrated no interaction between gender and occupations or between gender and sun protection habits (not shown in table). This proved that men was not bound to work outdoors and adopt sun protection measures infrequently. Similar confounding effects also happened to photoaging and history of cataract.

Education plays a pivotal role in the occurrence of SC. Admittedly, a good education qualification ensures us the enlightenment of regular physical examinations, evocation of self-skin examination and emphasis on early detection of SC^28^. Our outcome of multivariate analysis validated no significant decrease in the probability of SC among those with higher education. As we stated above, a proportion of subject in our survey were the bulk of the industrial workforce in the last century and were exposed to ultraviolet regardless of gender. Another clue could be that despite the fact that the majority were well educated and nearly all acknowledged the harm from prolonged sun exposure, sun protection habits during outdoor activities were still largely neglected. They were more concerned with dark spots, but less attentive to sunburn, scaly erythema and even SC. Therefore, strengthening and publicizing sunscreen consciousness is also the profound essence of this survey.

Skin phototypes were defined based on complexion, the degree of post-sun erythema, and sunburn by reference to Fitzpatrick system^29^. Available epidemiological data documented a preferential morbidity of SC in populations of skin phototypes I–II^30,31^. The pigmentary system generated by light-absorbing melanin biopolymers in melanocytes of epidermis serves as a visible marker of the skin’s defense against solar radiation^32^. Disappointingly, our data showed no significant distinction across all the phototypes, failing to support the previous documents. This might be due to the fact that Shanghai communities were generally homogeneous in terms of race and phototypes. 94.3% of the population had medium complexion, with very small percentages of light (2.4%) and dark (3.3%) skin tones.

Admittedly, multiple predisposing risks are involved in the etiology of skin malignancies^33^, and therefore SC vary vastly by geographical areas^4,34^. Outdoor workers are often exposed to high levels of UV radiation^35^. New Zealand^36^, France^37^, and Austria^38^ all reported high occupational UV exposure of farmers. The adverse effects of working outdoors is particularly relevant to possible photochemical damage to the skin and eyes. Examples are actinic keratosis, non-melanoma and malignant melanoma of the skin, and pterygium, cataract and macular degeneration of the eye. A meta-analysis by Bauer et al. revealed that outdoor workers had a 40% increased risk of BCC compared with indoor workers^39^. Our survey indicated a greater incidence of cutaneous tumors in the elderly who had experienced excessive outdoor work. Strangely, no beneficial effect from sun protection was supported by our findings. We believed this might be attributed to the interference of other factors, such as age and education. In addition, it also reflected the very weak differences in sun protection behaviors among Shanghai community residents, and only indoor and outdoor work could better elucidate the effects of UV rays on their skin. Other seemingly harmful factors such as eye wrinkles and inherited skin diseases did not exert any influence in this study. Further exploration will be conducted in a larger population sample in the future.

The exposed area of head and neck was the prone area of SC. In this study, AK and BC occupied 87.3% and 66.7% of head and neck SC respectively, which was close to previous studies^40,41^. These implied that AK and BCC might be more vulnerable to chronic UV exposure^34^. AK is acknowledged as a premalignant skin lesion that can evolve towards intraepidermal SCC, requiring prompt treatment^42,43^. A systematic review reported that the progression rate of AK to SCC was 0–0.075%^44^. In our investigation, AK exhibited a dominant position in all SC (80.8%).

A previous investigation asserted that AK affected nearly half of the global population (beyond 40%)^15^, far higher than our percentage. This was perhaps attributed to the majority of previous studies derived from clinical and pathological records rather than from large-scale surveys of healthy residents in a community. Another reason was thought to be that SC prevalence also varied by geographic locations, skin types, ethnicity or lifestyle.

We screened out 47 cases of SC with main comorbidities (For those complications overlapping in the same individual, the categorization was based on the most severe disease). Hypertension and diabetes constituted the largest proportion, representing 33.3% and 11.5% respectively. One meta-analysis once presented that calcium channel blockers and β-blockers users were at increased risk of developing SC and melanoma, respectively, owing to photosensitizing properties of these anti-hypertensive drugs^45^. The second dominant complication of SC was diabetes (9 cases). A retrospective cohort study in Taiwan reported that SC incidence was 3.2/10,000 person-years in the diabetes cohort, 1.18 times higher than that in the non-diabetic cohort^46^. The sustained hyperglycemia, high-level serum insulin and insulin-like growth factor were regarded as possible mechanisms for carcinogenesis in diabetic patients^47,48^. Additional investigations are needed for correlation mechanism between SC and its complications.

Almost every elderly person surveyed suffered from SK, which was in accordance with previous reports^49,50^. SK is usually considered as a sign of skin senility and the preferred areas where it appears are the exposed body-sites such as cheek and forehead^51^ (Supplementary Table S2 and Supplementary Fig. S1). Empirical evidence demonstrated that aging and long-term UV radiation were believed to be the dominant etiology for SK^52^. We calculated SK number at each age and discovered that senescence was positively correlated with SK number (Supplementary Fig. S2). These benign tumors were deemed to ruin aesthetics and be not harmful to health.

With regard to limitations to this study, one was the absence of an insight into the dynamic trend of ST incidence over successive years. As a matter of fact, we once attempted a follow-up to these objects in subsequent years. As we expected, there were many lost visits due to various reasons (such as visceral disease or death, especially those over the age of 80), resulting in an invalid statistics. Additionally, the accuracy and severity of these comorbidities could not be objectively assessed because they were self-reported based on the memory of the elderly. A further limitation was that although we surveyed a highly representative community, our study was restricted to one community. Therefore, this deficiency needs to be further improved in our future large-scale investigation of ST.

Despite above limitations, we performed the first epidemiological survey of epithelial tumors of the elderly in a community in Shanghai. Our constructive findings emphasized intensive propaganda work for early prevention and provided valuable reference for clinicians and public health authorities to guild early diagnosis and timely treatment.

## Supplementary Information

Supplementary Information.

## Data Availability

The datasets generated and/or analysed during the current study are not publicly available due to the confidentiality of data but are available from the corresponding author on reasonable request.
