# Supplementary material for: An epidemiological study on skin tumors of the elderly in a community in Shanghai, China
Source: Sci Rep. 2023 Mar 17;13:4441. doi: 10.1038/s41598-023-29012-1 (PMC10023674; doi:10.1038/s41598-023-29012-1)
Supplement: Supplementary file 1 — Supplementary Information. [file 41598_2023_29012_MOESM1_ESM.docx]

| **Concomitant diseases** | **n (%)** |
| --- | --- |
| Hypertension | 26 (33.3) |
| Diabetes mellitus | 9 (11.5) |
| Rheumatoid arthritis | 4 (5.1) |
| Psoriasis | 4 (5.1) |
| Chronic obstructive pulmonary disease | 3 (3.8) |
| Urticaria | 1 (1.3) |

**Table S1.** Concomitant diseases of skin cancer in the elders.

| **Body-site** | **Sex (P < 0.0001)** | | **Total, n (%)** |
| --- | --- | --- | --- |
|  | **Male, n (%)** | **Female, n (%)** |  |
| Hand back | 639 (83.3) | 1100（86.5） | 1739 (85.3) |
| Temporal | 654 (85.3) | 985 (77.5) | 1639 (80.4) |
| Cheek | 526 (68.6) | 920 (72.4) | 1446 (71.0) |
| Frontal | 408 (53.2) | 601 (47.3) | 1009 (49.5) |
| Nose | 208 (27.1) | 476 (37.5) | 684 (33.6) |
| Neck | 164 (21.4) | 214 (16.8) | 378 (18.5) |
| Jaw | 101 (13.2) | 189 (14.9) | 290 (14.2) |
| Chest | 130 (16.9) | 90 (7.1) | 220 (10.8) |
| Back | 26 (3.4) | 15 (1.2) | 41 (2.0) |
| Leg | 2 (0.3) | 7 (0.6) | 9 (0.4) |
| Abdomen | 4 (0.5) | 3 (0.2) | 7 (0.3) |
| Scalp | 3 (0.4) | 4 (0.3) | 7 (0.3) |
| Forearm | 2 (0.3) | 4 (0.3) | 6 (0.3) |
| Trunk | 3 (0.4) | 1 (0.0) | 4 (0.2) |
| Hip | 1（0.1） | 0 (0) | 1 (0.0) |
| Foot | 0（0） | 1 (0.0) | 1 (0.0) |

**Table S2.** Body-site distribution of seborrheic keratosis.

| **Benign skin tumors** | **Sex, n (%) (P = 0.3407)** | | **Age, n (%) (P = 0.6673)** | | | | | |
| --- | --- | --- | --- | --- | --- | --- | --- | --- |
|  |  |  |  |  |  |  |  |  |
|  | **Male** | **Female** | **60-64**  **years old** | **65-69**  **years old** | **70-74**  **years old** | **75-79**  **years old** | **80-84**  **years old** | **≥ 85**  **years old** |
|  |  |  |  |  |  |  |  |  |
| Surveyed population | 767 | 1271 | 524 | 291 | 309 | 511 | 297 | 106 |
| Benign tumor total | 21 (2.7, 95%CI: 1.6-3.9) | 46 (3.6, 95%CI: 2.6-4.6) | 19 (3.6, 95%CI: 2.0-5.2) | 11 (3.8, 95%CI:  1.6-6.0) | 9 (2.9,  95%CI:  1.0-4.8) | 13 (2.5, 95%CI: 1.2-3.9) | 10 (3.4, 95%CI: 1.3-5.4) | 1 (0.9,  95%CI: -0.9-2.8) |
| Pigmented nevus | 6 | 13 | 4 | 4 | 3 | 6 | 2 | 0 |
| Syringoma | 0 | 10 | 7 | 1 | 0 | 1 | 1 | 0 |
| Sebaceous  Gland  hyperplasia | 5 | 4 | 3 | 2 | 1 | 0 | 3 | 0 |
| Skin tag | 1 | 6 | 2 | 0 | 1 | 3 | 0 | 1 |
| Cherry angioma | 4 | 2 | 0 | 0 | 2 | 2 | 2 | 0 |
| Keloid | 1 | 1 | 0 | 1 | 0 | 1 | 0 | 0 |
| Epidermoid cyst | 1 | 1 | 0 | 1 | 0 | 0 | 1 | 0 |
| Adenoma sebaceum | 1 | 4 | 0 | 0 | 0 | 0 | 1 | 0 |
| Neurofibroma | 0 | 1 | 0 | 1 | 0 | 0 | 0 | 0 |
| Porokeratosis | 1 | 0 | 0 | 1 | 0 | 0 | 0 | 0 |
| Cavernous hemangioma | 0 | 1 | 0 | 0 | 1 | 0 | 0 | 0 |
| Nevus of ota | 1 | 0 | 1 | 0 | 0 | 0 | 0 | 0 |
| Congenital hairy naevus | 0 | 1 | 1 | 0 | 0 | 0 | 0 | 0 |
| Blue nevus | 0 | 1 | 1 | 0 | 0 | 0 | 0 | 0 |
| Spilus naevus | 0 | 1 | 0 | 0 | 1 | 0 | 0 | 0 |

**Table S3.** Prevalence of benign skin tumors except for seborrheic keratosis.


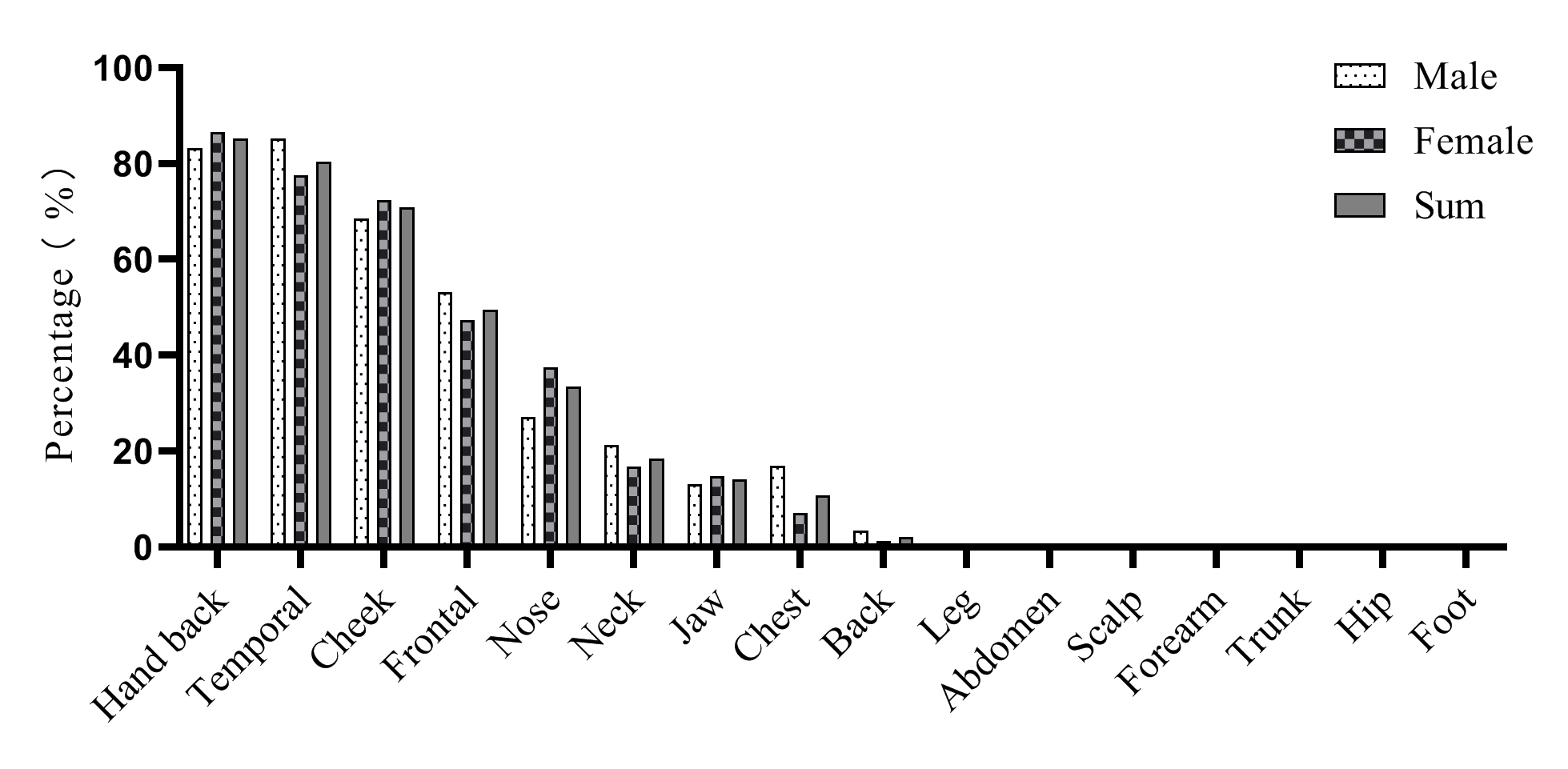


**Figure S1.** The body distribution of SK. The histogram showed SK prevalence varies greatly in different body sites in the elderly population. SK, seborrheic keratosis.


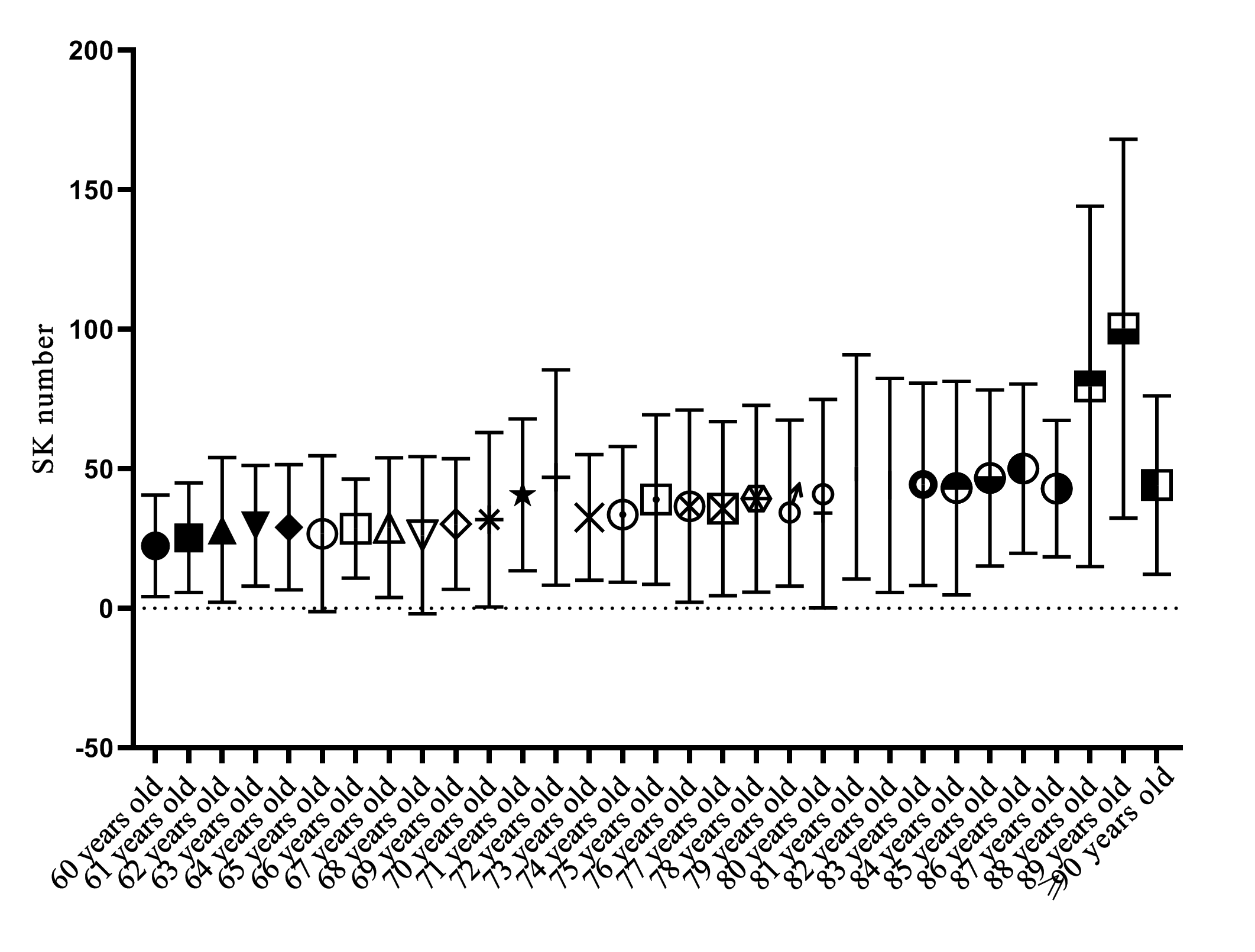


**Figure S2.** Correlation of the SK number and corresponding age. The figure displayed the occurrence of SK increased with age. SK: seborrheic keratosis.
